# Decitabine Induces Change of Biological Traits in Myelodysplastic Syndromes via FOXO1 Activation

**DOI:** 10.3389/fgene.2020.603956

**Published:** 2021-01-27

**Authors:** Zheng Zhang, Yan Jia, Feng Xv, Lu-xi Song, Lei Shi, Juan Guo, Chun-kang Chang

**Affiliations:** Department of Hematology, Shanghai Jiao Tong University Affiliated Sixth People’s Hospital, Shanghai, China

**Keywords:** myelodysplastic syndromes, DEGs, FOXO1, PTEN, TLR4

## Abstract

Decitabine (DAC) is considered to be a profound global DNA demethylation, which can induce the re-expression of silenced tumor suppressor genes. Little is known about the function of tumor suppressor gene FOXO1 in myelodysplastic syndromes (MDS). To address this issue, the study firstly investigated differentially expressed genes (DEGs) for DAC treatment in MDS cell lines, then explored the role of FOXO1 through silencing its expression before DAC treatment in MDS. The results showed that FOXO1 exists in a hyperphosphorylated, inactive form in MDS-L cells. DAC treatment both induces FOXO1 expression and reactivates the protein in its low phosphorylation level. Additionally, the results also demonstrated that this FOXO1 activation is responsible for the DAC-induced apoptosis, cell cycle arrest, antigen differentiation, and immunoregulation in MDS-L cells. We also demonstrated DAC-induced FOXO1 activation upregulates anti-tumor immune response in higher-risk MDS specimens. Collectively, these results suggest that DAC induces FOXO1 activation, which plays an important role in anti-MDS tumors.

## Introduction

Myelodysplastic syndrome (MDS) is a set of highly heterogeneous myeloid neoplasms, featured by variable cytopenias, inefficient hematopoiesis and a proportionable risk of progression to acute myeloid leukemia. The incidence of MDS in the elderly gradually increases with age, which is one of the main factors that threaten the quality of life and survival of the elderly. Although 3 therapies targeting MDS have been approved since 2004, the overall 5-year survival rate remains relatively poor at approximately 31% without a clear temporal improvement in outcomes (Zeidan et al., 2019). The heterogeneity of MDS requires a variety of complex and personalized treatments. Precision determination of prognosis is a key to select an suitable therapy and to forecast the prognosis of patients with MDS. According to the International Prognostic Scoring System (IPSS) or revised IPSS (IPSS-R), approximately 30% of patients are categorized as higher-risk groups (Greenberg et al., 1997, 2012). Hypomethylating agents (HMAs), such as decitabine (DAC), are approved as the first-line treatment option for higher- risk MDS. DAC has a wide range of therapeutic mechanisms for MDS, which reactivates silenced tumor suppressor genes, increases expression of cancer-testis antigens (CTAs), and regulates immune checkpoint molecules (Chang et al., 2017; Zhang et al., 2017a,b). Previous studies have shown that epigenetic silencing of tumor suppressor genes results in the growth advantage of a clonal subpopulation of MDS. This epigenetic modification is reversible, and methyltransferase inhibitors, such as DAC, will reverse the situation and reactivate the silenced tumor suppressor genes (Itzykson and Fenaux, 2014). The “O” subclass of the forkhead transcription factors (FOX) family is considered important tumor suppressor genes. FOXO genes exist widely in various organisms, they play an important role in the lifespan of invertebrates and mammals, and they inhibit tumor proliferation and regulate energy metabolism and induce cellular responses (Link and Fernandez-Marcos, 2017). In fact, a study has studied the effects of DAC treatment on the myeloid MDS cell line SKM-1 and investigated the role of FOXO3A in DAC-dependent treatment (Zeng et al., 2017). Currently, no studies have investigated the pathogenesis of FOXO1 in MDS.

FOXO1, also known as forkhead in rhabdomyosarcoma-like protein 1 (FKHRL1), is another key transcription factor of the FOX family with important roles in anti-oxidative enzymes, cell cycle arrest, apoptosis, autophagy, metabolic and immune regulators, which make it a super transcription factor with complex activities it a super transcription factor with complex activities (Murtaza et al., 2017; Jiang et al., 2018). It is characterized by the existence of a unique forkhead DNA binding domain, a highly conserved wing-helix motif, and regulation the transcription of a variety of downstream genes (Chen et al., 2019). The function of FOXO1 is not only regulated by microRNAs, which play important roles in destabilizing or attenuating the translation of FOXO1 mRNA, but also by post-translational modifications (such as phosphorylation, acetylation, and ubiquitination), which ultimately affect its nuclear/cytoplasmic transport and thus its cellular localization (Xing et al., 2018). After FOXO1 is phosphorylated, p-FOXO1 binds to the cytoplasm by 14-3-3 proteins and is involved in the subsequent interaction with ubiquitin E3 ligases, which induces its degradation, FOXO1 is inactivated and its target gene is down-regulated (Brownawell et al., 2001). Furthermore, FOXO1 is directly or indirectly regulated by other protein kinases (such as AKT, MAPK1, and PTEN). FOXO1 is considered as a potential tumor suppressor gene that participates in regulating the differentiation of a variety of cells and plays a role in inhibiting tumor cell proliferation (Ushmorov and Wirth, 2018). An increasing number of studies have confirmed that the re-expression and activation of FOXO1 in tumor cells has great potential in anti-tumor therapy (Shi et al., 2018).

In this study, we investigated the effects of a low concentration of DAC (1 μM) on differentially expressed genes (DEGs), cell apoptosis, cycle arrest, differentiation, and immunoregulation in MDS cell lines and patients. In addition, we also investigated the role of FOXO1 in DAC-dependent processes by measuring the expression level and activity of this gene and its downstream targets after DAC treatment.

## Results

### Transcriptome Profiling of MDS Cell Lines Following DAC Treatment

The GEM analysis of MDS-L cell showed 1,745 differentially expressed genes compared with the control: 842 were upregulated, while 903 genes were downregulated. The GEM analysis of SKM-1 cell showed 1,303 differentially expressed genes compared with the results of the control: 541 were upregulated, while 762 genes were downregulated. The overlap among the 2 cell lines contained 256 genes. Among them, 89 genes were upregulated together and 167 genes were downregulated together.

### GO Terms and Pathways Enriched by DEGs

**FIGURE 1:**
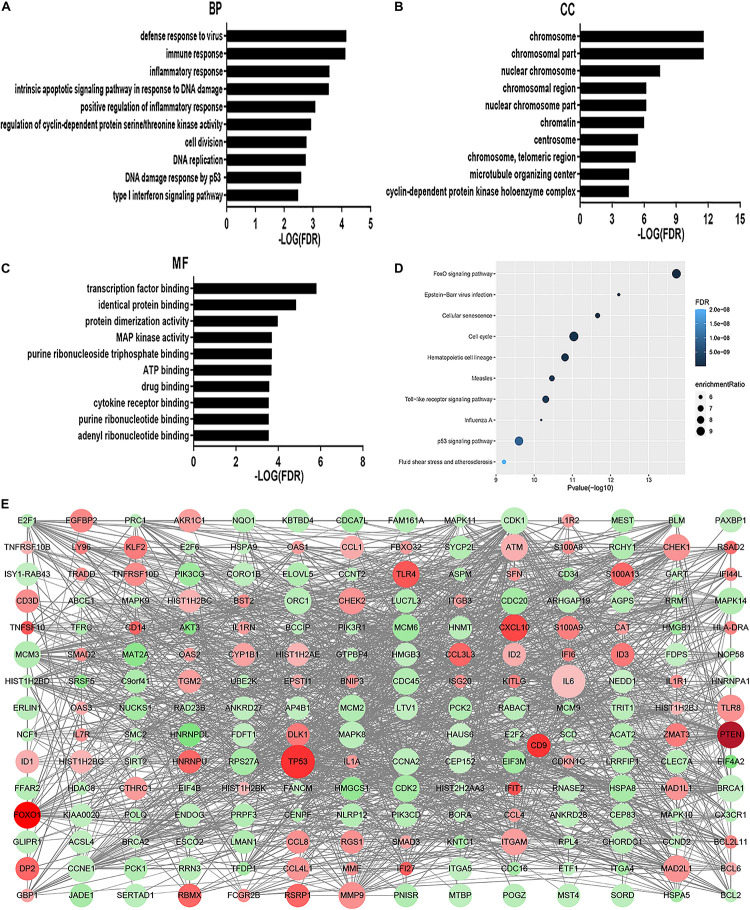
GO analysis and KEGG analysis. Results of BP **(A)**, CC **(B)**, and MF **(C)** in GO analysis revealed the relationship between hub genes and functional pathways. Top 10 pathway enrichment was shown by bubble chart **(D)** in KEGG analysis. The FDR < 0.05 was considered as significance. BP, biological process; CC, cellular component; MF, molecular function; KEGG, Kyoto Encyclopedia of Genes and Genomes. DEGs PPI network was constructed containing 256 DEGs based on the STRING online database (89 upregulated DEGs labeled in red and 167 downregulated DEGs labeled in green). The size of dots represents the node degree **(E)**.

The GO analysis revealed that MDS cell line DEGs are involved in the process of immune-related response. Biological process (BP) was mainly enriched in defense response to virus, immune response, inflammatory response, intrinsic apoptotic signaling pathway in response to DNA damage, and positive regulation of inflammatory response (Figure 1A). For cellular component (CC), enrichment of DEGs was mainly enriched inside the chromosome, chromosomal part, and nuclear chromosome (Figure 1B). For molecular function (MF), enrichment of DEGs was primarily in transcription factor binding, identical protein binding, and protein dimerization binding (Figure 1C). KEGG pathway analysis showed that DEGs enrichment occurred principally in the FOXO signaling pathway, Epstein-Barr virus infection, cellular senescence, cell cycle, and hematopoietic cell lineage (Figure 1D). Overall, there were significantly altered transcripts participating in the FOXO signaling pathway, cell cycle, Toll-like receptor signaling pathway, hematopoietic cell differentiation, inflammatory response, and p53 signaling pathway (Figure 1E).

### Functional Network of DAC-Induced Transcripts

**FIGURE 2:**
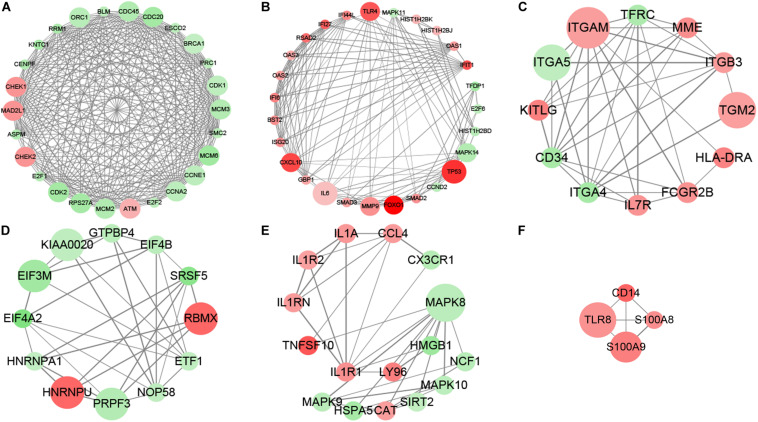
MCODE analysis. MCODE analysis based on the degree of importance, and six main subnetworks were displayed. Upregulated genes are marked in red; downregulated genes are marked in green. The size of dots represents the node degree. **(A–F)** Showed the six main subnetworks using MCODE analysis.

The subnetwork enrichment analysis of DAC induced immune related transcripts in MDS cell lines identified TP53, FOXO1, TLR4, TLR8, S100A8, S100A9, CD14, and CXCL10 as highly interconnected genes, and are likely to be the potential hubs of the immunity functional network (Figure 2). The FOXO1 signaling pathway containing genes AKT3, ATM, BCL2L11, BCL6, BNIP3, CAT, CCND2, CDK2, FBXO32, FOXO1, HOMER1, IL6, IL7R, KLF2, MAPK11, MAPK14, MAPK8, MAPK9, PCK1, PCK2, PIK3CD, PIK3CG, PIK3R1, PTEN, SMAD2, SMAD3, and TNFSF10 are biologically linked to numerous signal pathways, including cell apoptosis, cell cycle, cell differentiation, and immune system. In this study, we concentrated especially on the effect of FOXO1 on biological characteristics in MDS.

### DAC Induces Apoptosis in MDS-L Cells

**FIGURE 3:**
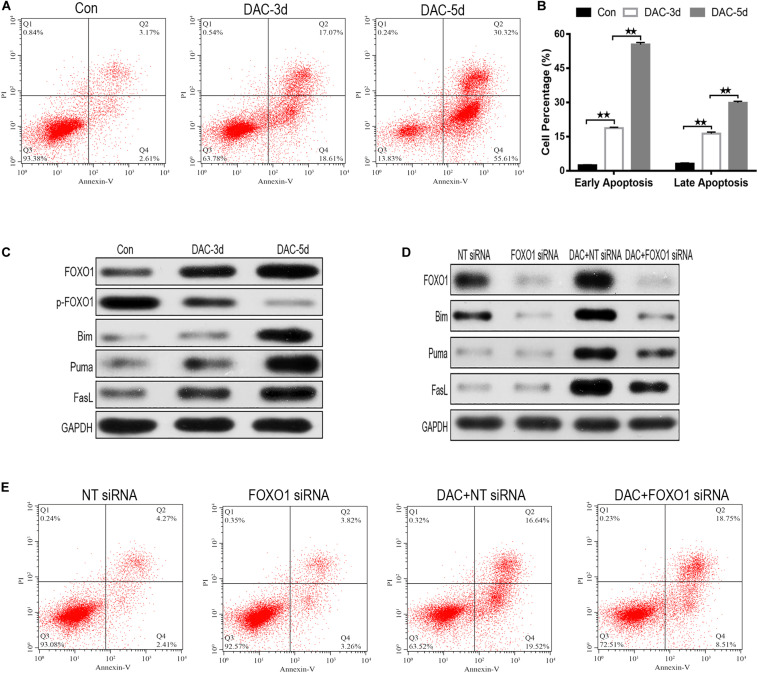
Decitabine (DAC) induces apoptosis in MDS-L cells. Annexin-V-FITC/PI assay showed that apoptosis occurred in MDS-L cells with DAC prolonged exposure time **(A)**. FCM assay detected that DAC treatment could induce both early and late apoptosis in MDS-L cell **(B)**. Western blot found that FOXO1, Bim, Puma, and FasL in MDS-L cells increased with p-FOXO1 protein decreasing after DAC treatment **(C)**. FOXO1 silencing had no obviously influence on Puma and FasL, but decreased the expression of Bim that was observed following DAC treatment **(D)**. MDS-L cell early apoptosis was partly inhibited after FOXO1 silencing and could not be significantly induced by subsequent DAC treatment **(E)**. **Student’s *t*-test *P* < 0.01.

As DAC exposure time expanded, the percentages of apoptotic cells increased significantly (Figure 3A). Annexin-V-FITC/PI double labeling confirmed the rate of apoptosis. The labeling showed a gradual increase in apoptosis on days 3rd and 5th day, confirming that with the increase in early apoptotic cells is the most significant rate (Figure 3B).

In the absence of DAC, the expression of activated FOXO1 was very low in MDS-L cells, but after the initiation of treatment on days 3 and 5, the expression of activated FOXO1 gradually increased, rather than the non-activated phosphorylated form (p-FOXO1). The expression of p-FOXO1 gradually decreased, indicating that FOXO1 mainly exists in an inactive form in MDS-L cells. With the prolonged action of the drug, DAC can induce FOXO1 activation in MDS-L cells (Figure 3C). The expression of target protein downstream of apoptosis-related FOXO1 was also detected. As shown in Figure 3C, measurable expression of apoptosis-related proteins Bim, Puma, and FasL was observed in untreated MDS-L cells. After DAC treatment, Bim, Puma, and FasL protein expression increased significantly with the increase of exposure time to drug (Figure 3C).

To investigate the role of FOXO1 in DAC-induced MDS-L cell apoptosis, we suppressed FOXO1 expression by targeting siRNA before DAC treatment. Western blot displayed that siRNA targeting FOXO1 decreased FOXO1 expression approximately 72% compared to negative control siRNA. After DAC treatment, FOXO1 expression increased obviously in negative control siRNA-treated MDS-L cells. In contrast, the expression of FOXO1 showed no significant increase when cells were treated with FOXO1-targeted siRNAs (Figure 3D). These data confirm that FOXO1 expression is inhibited by siRNA. We also observed that silencing FOXO1 expression helped to suppress Bim expression after DAC treatment, but not the expression of Puma and FasL protein, suggesting that the presence and activation of FOXO1 plays a crucial role in the activation of Bim (Figure 3D).

After knockdown of FOXO1, apoptosis assay indicated that silenced FOXO1 did not significantly affect the later apoptosis of MDS-L cells, but significantly decreased early apoptosis of MDS-L cells, suggesting that FOXO 1 activation is involved mostly in the early stages of DAC-induced apoptosis (Figure 3E).

### DAC Induces Cell Cycle Arrest in MDS-L Cells

**FIGURE 4:**
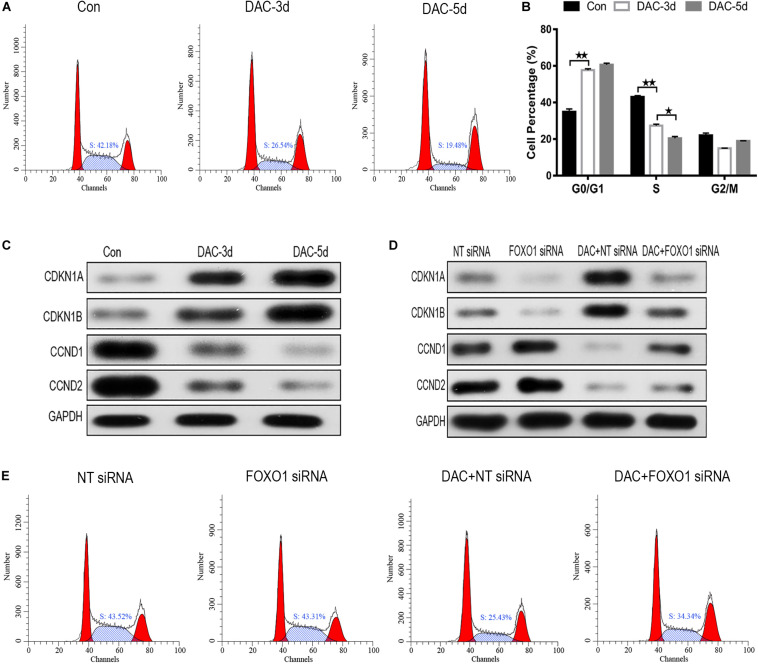
Decitabine induces cell cycle arrest in MDS-L cells. Annexin-V-FITC/PI assay showed that DAC treatment could decrease cells in S phase and arrest MDS-L cells in G0/G1 and G2/M phases **(A,B)**. Western blot found that CDKN1A and CDKN1B in MDS-L cells increased with CCND1 and CCND2 protein decreasing after DAC treatment **(C)**. FOXO1 silencing had no obviously influence on CDKN1B and CCND1, but decreased the expression of CDKN1A and CCND2 that was observed following DAC treatment **(D)**. DAC-induced reduction of MDS-L cells in the S phase was significantly attenuated after silencing FOXO1 **(E)**. **Student’s *t*-test *P* < 0.01 and *P < 0.05.

After DAC treatment, the ratio of cells in S phase decreased significantly, while the proportion of cells in G0/G1 phase increased, indicating that cell cycle arrest was induced by G0/G1 blockade (Figures 4A,B). The influence of DAC treatment on cell cycle gene expression was also observed. CDKN1A, CDKN1B, CCND1, and CCND2 are downstream genes targeted by FOXO1 and are disordered in a multiple of tumors. As shown in Figure 4C, the expression of CDKN1A and CDKN1B was scarce in untreated MDS-L cells, but after DAC treatment, the expression of CDKN1A and CDKN1B was upregulated with drug maintenance application. Conversely, a significant decrease in CCND1 and CCND2 expression was observed in the presence of DAC (Figure 4C).

Then, we studied the effect of silent FOXO1 on CDKN1A, CDKN1B, CCND1, and CCND2. Compared with control siRNA, silencing FOXO1 had significant effects on the cell cycle. The expression of FOXO1 downstream targets CDKN1A and CCND1 were obviously affected, whereas silencing FOXO1 had no significant effect on CDKN1B and CCND2 (Figure 4D). In the presence of FOXO1 silencing, DAC showed no obviously ability to regulate either CDKN1B and CCND2 protein expression, indicating FOXO1 has a regulatory effect to CDKN1A and CCND1 (Figure 4D). The increase in the proportion of S phase cells in MDS-L following knockdown of FOXO1 was not totally reversed by subsequent DAC treatment, suggesting that FOXO1 activation plays an indispensable role in DAC-induced cell cycle arrest (Figure 4E).

### FOXO1 Contributes to DAC-Induced MDS-L Cell Differentiation

**FIGURE 5:**
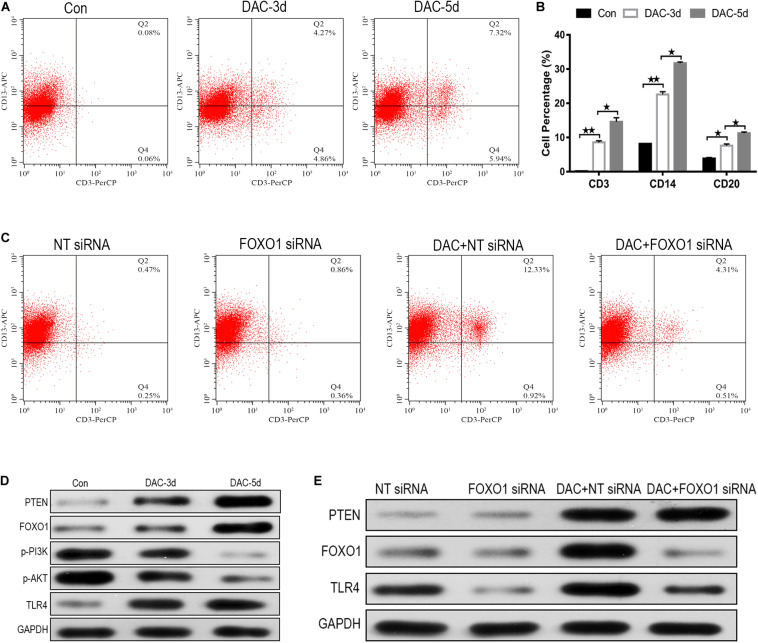
FOXO1 contributes to DAC-induced MDS-L cell differentiation. CD3 expression on CD13 was significantly induced in MDS-L cells treated with DAC for different action times **(A)**. The percentage of CD13 expressing CD3, CD14, and CD20 antigen increased following DAC treatment **(B)**. Surface CD3 and CD13 expression had clearly no change between FOXO1 siRNA and negative control siRNA in MDS-L cells, while surface CD3 expression was impaired in FOXO1 siRNA compared with negative control siRNA in DAC-treated MDS-L cells **(C)**. Western blot showed that PTEN, FOXO1, and TLR4 in MDS-L cells increased with p-PI3K and p-AKT protein decreasing after DAC treatment **(D)**. FOXO1 silencing had no obviously influence on PTEN, but decreased the expression of TLR4 that was observed following DAC treatment **(E)**. **Student’s *t*-test *P* < 0.01 and *P < 0.05.

MDS-L cells were positive for CD34, c-Kit, HLA-DR, CD13, and CD33, and partially positive for CD41 and negative for CD3, CD14, CD20, and CD235a (Tohyama et al., 1994). The expression levels of myeloid cell antigen CD13, T lymphocyte cell marker CD3, monocyte differentiation marker CD14, B lymphocyte differentiation marker CD20, and erythroid cell differentiation marker CD235a on the surface of DAC treated MDS-L cells were detected. The expression levels of CD3, CD14, and CD20 on the surface of MDS-L cells increased after DAC treatment, accompanied by antigen changes, with CD13 showing a significant decrease after treatment, while the expression of CD235a showed no obvious change during DAC treatment. As DAC action time was prolonged, the expression levels of CD3, CD14, and CD20 continued to increase in a time-dependent manner (Figures 5A,B).

No significant difference in cell differentiation antigen expression was observed between MDS-L cells in which FOXO1 was the silenced and non-silenced control. However, when FOXO1 siRNA-MDS-L cells were treated with DAC, the observed increase in CD3-positive cells were significantly reduced compared to cells bearing negative control siRNA (Figure 5C), indicating that silencing FOXO1 before DAC treatment weakens, but does not eliminate the differentiation of DAC-induced MDS-L cells into antigen molecules. Therefore, the above studies indicate that FOXO1 activation contributes to DAC-induced MDS cell differentiation.

### FOXO1 Contributes to DAC-Mediated TLR-4 Augment in MDS-L Cells

The PTEN/PI3K/AKT/FOXO1 signaling pathway is a major signaling pathway involved in cell proliferation, apoptosis, metastasis, and immunoregulation, and its cascade reaction pathway occupies an important position in the signal transduction process (Jiang et al., 2018). In this study, the protein expression of PTEN, FOXO1, p-PI3K, p-AKT, and TLR-4 was also detected employing western blot. The results showed that the protein expression of PTEN, FOXO1, and TLR-4 increased after DAC treatment, accompanied by significant decreases in p-PI3K, and p-AKT (Figure 5D). Compared with the negative control group, FOXO1 siRNA-MDS-L cells treated with DAC, the expression of PTEN showed significant upregulation, but the expression of FOXO1 and TLR-4 showed a significant decrease (Figure 5E). Thus, DAC induces PTEN, which in turn activates FOXO1 signaling, leading to the activation TLR4-driven innate immune response.

### FOXO1 Contributes to DAC-Mediated Immune Activation in MDS Patients

**FIGURE 6:**
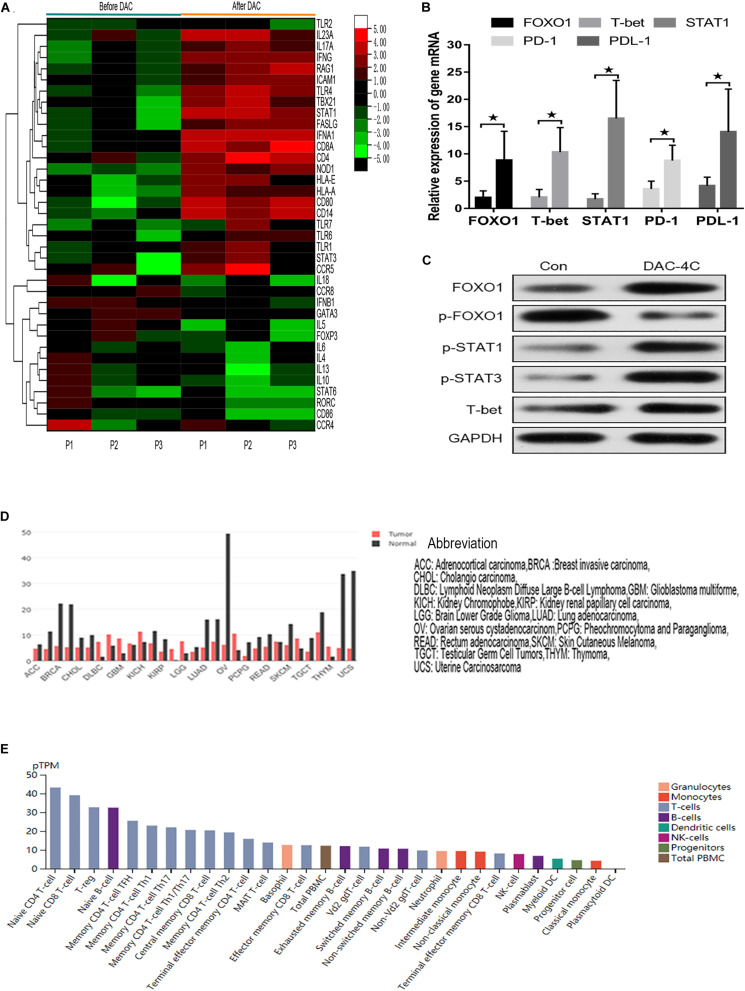
Hierarchical heat map showing the gene DEGs in immune processes after DAC treatment. Normalized log_2_ transformed values as determined by RT^2^ Profiler Human Innate and Adaptive Immune Responses PCR Array in T lymphocytes of 3 matched MDS patients collected at non-DAC and 4-course-DAC treatment. Each column represents one MDS patient, each horizontal line refers to one gene. The cutoff value of log fold change as >1.5 or <−1.5 and false discovery rate <0.01 was considered **(A)**. The bar charts showed that expression of FOXO1, T-bet, STAT1, PD-1, PDL-1 increased significantly after DAC treatment (B). Western blot showed that FOXO1, p-STAT1, p-STAT3, and T-bet in one MDS patient increased with p- FOXO1 protein decreasing after DAC treatment **(C)**. Using the GEPIA (Gene Expression Profiling Interactive Analysis) dataset (http://gepia.cancer-pku.cn/), we compared the mRNA expression of FOXO1 between different tumors and normal tissues. The results indicated that the expression levels of FOXO1 were obviously higher in normal tissues than in tumors. The gene expression profile across different tumor samples and paired normal tissues (Bar plot). The height of bar represents the median expression of certain tumor type or normal tissue **(D)**. Utilizing an online tool (The Human Protein Atlas which aim to map all the human proteins in cells, tissues and organs using integration of various omics technologies, https://www.proteinatlas.org/), we explored the mRNA expression of FOXO1 in immune cells. The results show FOXO1 plays an important role in immune cell development **(E)**. * means *P* < 0.05.

Because MDS-L lacks innate and adaptive immune cells, MDS patient specimens were used to verify the effect of FOXO1 on innate and adaptive immunity *in vivo*. The transcriptional profiling of 84 genes involving innate and adaptive immune processes were evaluated after 4 courses DAC treatment in 3 matched MDS patients (*n* = 3) (Supplementary Table 1). The transcriptional profiling analysis was performed on isolated T cells. A total of 37 (44%) genes were differentially expressed after DAC treatment with fold changes >2.5 (Figure 6A). Among these, a total of 23 (27.4%) innate and adaptive immunity genes were significantly upregulated. The altered transcriptional profiling of MDS T cells was characterized by the upregulation of innate and adaptive immunity genes. Meanwhile, the expression of FOXO1, STAT1, T-bet, PD-1, and PD-L1 was also detected in 12 paired MDS patients by RT-PCR due to the limiting amount of the panel. As shown in Figure 6B, the mRNA level of FOXO1, T-bet, STAT1, PD-1, and PD-L1 was highly upregulated after 4 courses of DAC treatment. Furthermore, the protein expression level of FOXO1, p-FOXO1, p-STAT3, and T-bet was detected by western blot in 1 MDS patient. As shown in Figure 6C, the expression of activated FOXO1, p-STAT1, p-STAT3, and T-bet increased, while the expression of non-activated p-FOXO1 reduced after DAC treatment. Therefore, the above studies demonstrate that FOXO1 activation contributes to DAC-induced immune activation in MDS.

## Discussion

Myelodysplastic syndromes indicates that the status of pre-leukemia with ineffective hematopoiesis, featured by bone marrow dysplasia that easily progresses into acute myeloid leukemia (Steensma, 2018). For higher-risk MDS patients who are not suitable for transplant, HMAs are most appropriate. DAC are therapeutic agents and have already been used in treatment of higher-risk MDS and acute myeloid leukemia for many years. DAC can play a role in decreasing MDS clonal burden, then resulting in improved hematopoiesis, but do not eradicating tumor stem cells, so relapse is inevitable tendency. The mechanism of action of DAC is still not fully understood, and may result from a combination of conventional cytotoxic, DNA hypomethylation and immune-related mechanisms including changes in interferon signaling and presentation of neoantigens as epitopes to the immune system (Licht, 2015; Chiappinelli et al., 2017). Once DAC fails due to intolerance, resistance, or relapse after a favorable response, the approved second-line therapy is limited, the outlook is poor, and the median survival time is less than 6 months (Jabbour et al., 2010; Prébet et al., 2011; Montalban-Bravo et al., 2018). Therefore, clarifying the mechanism underlying DAC is required to improve the treatment effect in MDS.

Decitabine is considered as a rapid and profound global DNA demethylation, as well as site-specific promoter demethylation of many genes including cancer suppressor genes and immune-related genes (Wolff et al., 2017; Seelan et al., 2018). In this study, using the DEG analytical method, the data suggest that multiple genes and multigenic pathways were regulated by DAC. For example, DAC could activate the interferon signaling and p53 signaling pathway induce further biological process in pathway analysis as previous reports (Chang et al., 2017; Zhang et al., 2017a,b). However, this study focused on the mechanism of tumor suppressor gene FOXO1 in DAC -dependent processes. The results indicated that FOXO1 is hyperphosphorylated in MDS-L cells and thus inactivated. DAC treatment activates FOXO1 by increasing its gene expression and reducing its protein phosphorylation, leading to up-regulation of downstream effectors Bim, Puma, FasL, CDKN1A, and CDKN1B and down-regulation of downstream cell cycle effectors Cyclin D1 and Cyclin D2. Furthermore, DAC-induced differentiation of MDS-L cells into lymphocytes and monocytes, MDS-L cell cycle arrest, apoptosis, and immune activation were also observed.

Forkhead box O (FOXO) transcription factors, including FOXO1 (FKHR), FOXO3a (FKHRL1), FOXO4 (AFX), and FOXO6, have also been increasingly recognized as tumor suppressors through serving as pivotal connection points to allow stress signals, proliferative nutrient and diverse to cluster and integrate with distinct gene networks to control cell fate, metabolism, and cancer development (Farhan et al., 2017; Jiramongkol and Lam, 2020). As shown in Figure 6D, FOXO1 expression is reduced in most tumors and appears to act as a tumor suppressor gene. In this study, similar to the effect of FOXO3A activation in the SKM-1 cell line (Zeng et al., 2017), active FOXO1 also plays an important role in suppressing MDS-L by inducing apoptosis and by supressing progression of cell cycle. In contrast to active FOXO3A, our research confirmed that activated FOXO1 tends to directly regulate Bim gene, which is consistent with previous research (Dijkers et al., 2000, 2002). That is, activated FOXO1 is translocated to the nucleus, binding to the Bim promoter and induces the transcription of the Bim gene. However, FOXO1 activation showed a limited regulation effect on Puma and FasL expression by silencing FOXO1, which shows that DAC may up-regulate the expression of Puma and FasL in other ways. Therefore, it is possible that the apoptosis induced by DAC is mainly mediated through the mitochondrial apoptosis pathway. Through a similar silencing FOXO1 gene function test, we also found that FOXO1 has a more closely regulatory effect on CDKN1A and CCND2 than on CDKN1B and CCND1 (Schmidt et al., 2002).

Next, we focused on the role of FOXO1 in the MDS immune microenvironment. As shown in Figure 6E, FOXO1 also inherently controls the anti-tumor immune response and the homeostasis and development of immune cells, including T cells, B cells, natural killer (NK) cells, macrophages, and dendritic cells. However, the mechanism of FOXO1 in the MDS immune environment has not been reported so far. In the past decade, aberrant immune activation in lower risk MDS and impaired anti-leukemic immunity in higher risk Malignant clones as well as MDS in the bone marrow microenvironment were identified as key pathogenic drivers of MDS. Multiple mechanisms are involved in higher risk MDS to promote immune tolerance (Ivy and Brent, 2018; Sallman and List, 2019). Several reports have demonstrated that higher risk disease is accompanied by an increase in myeloid-derived suppressor cells (MDSCs), along with regulatory T cells (Tregs) augmentation (Gabrilovich and Nagaraj, 2009; Kittang et al., 2015). One effect of increasing suppressive cell subsets is to reduce cytotoxic anti-leukemia immunity. The activation and function of NK cells and cytotoxic T lymphocytes (CTL) play important cytotoxic activity in response to myeloid neoplasms, but their functions are reduced in high-risk MDS (Epling-Burnette et al., 2007; Kotsianidis et al., 2009). In our study, the results showed that DAC promotes the differentiation of MDS-L cell surface antigen into lymphocytes and monocytes through activating FOXO1. Further PTEN/PI3K/AKT/FOXO1 pathway study confirmed that DAC upregulates PTEN gene expression, thereby inhibiting the phosphorylation of PI3K and AKT and releasing FOXO1 inhibition. Activated FOXO1 can upregulate TLR4 expression, thereby activating the MDS innate immune system. The results were consistent with previous studies, FOXO1 is a key regulator of many inflammatory factors, such as TRL4, NF-κB, tumor necrosis factor (TNF)-α, interleukin (IL)-1β, and IL-18, via the TLR4/NF-κB signaling pathway (Kamo et al., 2013; Li et al., 2016). In the specimens of MDS patients, we further verified that with the increased expression of FOXO1, there is a wide range of upregulation and activation of type I immune cell transcription factors and up-regulation of cellular immune functions, thereby enhancing anti-leukemia effects and inhibiting the growth of MDS malignant clones. All the results demonstrated that DAC can activate FOXO1 to enhance anti-tumor immune effect in higher risk MDS.

Accumulating evidence has shown the paradoxical intrinsic role of the FOXO1 in cancer, which can act as a tumor repressor while also maintaining cancer stem cells (Sykes et al., 2011; Long et al., 2020). The regulatory role of FOXO1 in tumor immunity is equally confusing (Deng et al., 2018). DAC also has a double-edged sword effect on the regulation of MDS immune function. The global demethylation of DNA can induce anti-tumor effects. It can also upregulate the expression of inhibitory immune checkpoint receptors and their ligands, resulting in secondary resistance to DAC (Wolff et al., 2017). Due to the limitation of conditions, we do not have a suitable animal model of MDS to further verify activated FOXO1 and their extracellular factors in cancer cells, stromal cells, and immune cells. These factors have a profound effect on promoting or inhibiting anti-cancer immunity in the tumor microenvironment. The effect of activated FOXO1 on the prognosis and survival of MDS disease also requires further long-term follow-up. Based on the above findings, we agree with assumption of Wolff F, who proposed a role for FOXO1 as a rheostat, which regulates both immune homeostasis and the immune response in cancer immunity. In the future, further study is needed to better understand the role of FOXO1 in MDS pathogenesis. It will be interesting to better understand each molecule and each cell type regulated by FOXO1 directly in the settings of cancer patients or tumor models (Tirosh et al., 2016).

In conclusion, this study showed that silencing FOXO1 expression impaired DAC-induced apoptosis, cell cycle arrest, and cellular differentiation, which potentially may be due to the observed down-regulation of Bim, CDKN1A, and CCND1. DAC-induced TLR4 upregulation was mainly reversed by FOXO1 silencing, which could explain the partial reversal of DAC-induced immune activation observed when FOXO1 expression was knocked down. It was found that the up-regulation of innate and adaptive immunity genes after DAC treatment, as well as the subsequent increase in T-bet, p-FOXO1, and p-STAT3 in MDS samples, were related to the up-regulation and activation of FOXO1 induced by DAC. Collectively, DAC induces FOXO1 activation, which plays an important role in anti-MDS tumors.

## Materials and Methods

### Cell Culture and DAC Treatment

MDS-L cells were donated by Prof. Tohyama (Tohyama et al., 1995). MDS-derived leukemia cell line SKM-1 cells were donated by Prof. Nakagawa (Nakagawa and Matozaki, 1995). Cell lines were cultured in RPMI 1640, which contained 10% heat-inactivated fetal bovine serum and 1% penicillin-streptomycin at 37°C in a humidified atmosphere containing 5% CO_2_. Furthermore, IL-3 (100 U/ml) is essential for MDS-L cells. When cells reached the logarithmic growth phase, they were seeded at a density of 5 × 10^5^ cells/well in 6-well plates and harvested with five 24-h pulses of 1 μM DAC (Selleck Chemicals LLC, Houston TX, United States) and collected after the treatment (Zhang et al., 2017b).

### RNA Preparation and Gene Expression Microarray (GEM)

As previously described, according to the manufacturer’s instructions, total RNA was extracted from 10^5^ cell lines using the RNeasy system (Qiagen, Valencia, CA, United States). A Genechip Primeview^TM^ Human Gene Expression Array (Affymetrix, United States) was used for the GEM study. The signal intensities were obtained with a Genechip Scanner 3000 7G (Affymetrix) to generate cell intensity files (CEL). Statistical analysis was performed using Partek Genomics Suite software (Partek, Inc., St. Louis, MO, United States). The robust multiple array average (RMA) algorithm was used to normalize the data. The false discovery rate (FDR) was lower than 0.15 to minimize the misidentification of genes. Analyzed the changes of up-regulated or down-regulated genes greater than 1.0 times (Xu et al., 2014).

### Functional Enrichment Analysis

WebGestalt^1^ is an automated systematic-analysis tool that helps understand common and unique way within a set of orthogonal target discovery researches. The tool is free and well-maintained, user-friendly gene-list analysis for gene annotation and analysis. The gene ontology (GO) terms for biological process (BP), cellular component (CC), and molecular function (MF) categories, as well as Kyoto Encyclopedia of Genes and Genomes (KEGG) pathways was performed using the WebGestalt online tool. GraphPad (GraphPad Software, Inc., San Diego, CA, United States) was used to improve GO graphic analysis. Fisher’s exact test was used to select the important approach, and the significance threshold was defined by FDR. *P*-value < 0.05 was considered statistically significant. STRING^2^ was employed to construct protein–protein interaction (PPI) network of DEGs for DAC treatment MDS cell lines and the confidence score of the interaction > 0.4 was considered statistically significant. Cytoscape was employed to annotate and improve the up-down regulated genes of PPI. Further, molecular complex detection (MCODE) APP was applied to identify densely connected network components (Xu et al., 2014).

### Cellular Transfection

MDS-L cells were transfected with either Silencer Select FOXO1 (cat. AM16708) or Silencer Select Negative Control (cat. AM4611) siRNAs, both of which were purchased from Thermo Fisher (Thermo Fisher Scientific, Waltham, MA, United States), and Lipofectamine 3000 reagent (Thermo Fisher Scientific, Waltham, MA, United States) according to previously reported literature (Zeng et al., 2017). The harvest time of these transfected cells was set to 24 h after transfection. Then, the transfected cells were either cultured or treated with 1 μM of DAC as needed for 72 h.

### Apoptosis Assessment

Cell apoptosis analysis was fulfilled by employing an annexin V-FITC/PI apoptosis detection kit (BD Biosciences) with a flow cytometer (FACS Calibur, BD Biosciences, Franklin Lakes, NJ, United States). According to different experimental manipulation, MDS-L cells were seeded and inoculated in a 6-well plate at a density of 10^5^ cells per well. Subsequently, these cells were first suspended in the binding buffer, and then Annexin V-FITC (10 μl) and PI (5 μl) were, respectively, supplied to each well. Finally, The mixture was reacted in the dark for 15 min and then analyzed using flow cytometry (FCM) (Zhang et al., 2017b).

### Cell Cycle Analysis

Wash 5 × 10^4^ cells with cold phosphate-buffered saline (PBS), fix in 70% ethanol, wash once with PBS, and then re-suspend in 1 mL of propidium iodide (PI) staining reagent (50 mg/ml of propidium iodide and 1 mg/ml of RNAse). Before performing cell cycle analysis, the samples were incubated in the dark for 30 min. The cell cycle was measured with FACS Calibur. The percentages of cells in the G1, S, and G2 phases were calculated with the Cellquest software (Xu et al., 2014).

### Detection of Cell Surface Markers

DAC-treated MDS-L cells were collected and stained for surface antigen, 10 μl of PerCP-bound anti-CD3 antibody, APC-bound anti-CD13 antibody, FITC-bound anti-CD14 antibody, PE-bound anti-CD20 antibody or PE-bound anti-235a antibody (Becton Dickinson) was added to the cells and incubated for 15 min at room temperature in the darkness. Cells were then washed and resuspended in PBS, and surface markers were analyzed by FCM within 1 h.

### Bone Marrow Mononuclear Cells and T Lymphocytes Preparation

Twelve MDS patients were administered with DAC (product name: Dacogen, DAC, Xian Janssen Pharmaceutical Ltd., 20 mg/M^2^ × 5 days for 4 courses) for immune research after obtaining informed consent according to the Declaration of Helsinki and Council for International Organizations of Medical Sciences International Ethical Guidelines, and ethical approval was obtained from the ethics review board at The Sixth Hospital Affiliated with Shanghai Jiao Tong University. All participants provided written informed consent prior to enrollment, and the privacy rights of human subjects were observed. MDS were diagnosed in accordance with the World Health Organization (WHO) 2016 criteria (Arber et al., 2016), and the detailed information regarding MDS patients is shown in Supplementary Table 1. Five milliliters of fresh bone marrow were aspirated from posterior iliac crests. Bone marrow mononuclear cells (BM-MNCs) were isolated using Ficoll-Hypaque gradient (Lymphoprep TM, Niergaard, Oslo, Norway) for mRNA expression by quantitative real-time polymerase chain reaction (PCR). BM-MNCs of some MDS patients were carefully separated into 2 portions, T cells of 1 part were isolated using MACS CD3+ T microbeads (Miltenyi Biotec), followed by RT^2^ Profiler PCR Arrays and the rest of the BM-MNCs were used for western blot analysis (Zhang et al., 2017b).

### Western Blot Analysis

Whole cell lysates were obtained from MDS-L cell line or BM-MNCs of MDS patients, and separate the same amount of protein by 12% sodium dodecyl sulfate polyacrylamide gel electrophoresis (SDS-PAGE), and then blotted onto a polyvinylidene difluoride (PVDF) membrane. The PVDF membranes were blocked with Tris-buffered saline (TBS) containing 5% skimmed milk powder for 1 h, and then incubated with the primary antibodies (Supplementary Table 2) at 4°C overnight. Membranes were incubated with either anti-mouse or anti-rabbit immunoglobulin G (IgG) horseradish peroxidase-conjugated secondary antibody (Amersham Biosciences, Piscataway, NJ, United States). Specific bands were visualized using ECL Western Blotting Detection Reagents (Amersham Biosciences, Piscataway, NJ, United States). The intensity of bands was quantified using Image Lab software version 2.0 (Bio-Rad Laboratories, Hercules, CA, United States), and glyceraldehyde 3-phosphate dehydrogenase (GAPDH) was used as an internal standard (Xu et al., 2014).

### RT^2^ Profiler PCR Arrays

According to the manufacturer’s instructions, 3 matched MDS patients were isolated from 3 × 10^6^ total T cells RNA using miRNeasy Micro Kit (Qiagen). RNA concentration and quality were evaluated by using Nanodrop-ND-1000 (Celbio). According to the manufacturer’s instructions, cDNA was synthesized from 250 ng of total RNA using RT^2^ First Strand Kit (SABiosciences Corp.), and the expression levels of 84 genes (Supplementary Table 3) were analyzed by RT^2^ Profiler Human Innate and Adaptive Immune Response PCR Array (PAHS-052Z, SABiosciences Corp). Real-Time PCR amplification was performed on a 7500 Real-Time PCR System (Applied Biosystems). The online tool RT^2^ Profiler data analysis software (Qiagen) was used for data standardization and statistical analyses. The threshold cut-off point was based on >2.5-fold differential expression. The Pheatmap package was used to investigate the differential expression of immune genes through PCR Arrays (Serena et al., 2018).

### Quantitative Reverse-Transcription Polymerase Chain Reaction (qRT-PCR)

Total RNA was extracted from BM-MNCs of 12 matched MDS patients using the RNeasy Mini Kit (QIAGEN, Hilden, Germany) according to the manufacturer’s instructions. Afterward, reverse transcription and qRT-PCR were, respectively, fulfilled by utilizing a Revert-AidTM First Strand cDNA Synthesis Kit (Fermentas, Burlington, ON, Canada) and SYBR Premix Ex TaqTM II (Tli RNaseH Plus) (TAKARA, Beijing, China). The threshold cycles were used to calculate relative expression levels according to the 2^–ΔΔCt^ method. Relative mRNA expression levels were normalized to the level of GAPDH. The primer sequences used in this investigation are shown in Supplementary Table 4 (Zhang et al., 2017a).

### Statistical Analysis

All experiments were performed at least 3 times. The analysis of flow cytometry data was performed using CellQuest software. Continuous variables were expressed as mean ± standard deviation (SD) or median. According to the distribution of each variable, the comparison was made according to parameters [Student’s *t* test, analysis of variance (ANOVA)] or non-parametric (Mann–Whitney *U* Test, Kruskal–Wallis). Categorical variables were compared using Fisher’s exact tests. The difference was considered statistically significant at *P* < 0.05. Statistical analyses were performed using GraphPad Prism program (GraphPad Software, Inc., San Diego, CA, United States) or SPSS software (version 22.0).

## Data Availability Statement

The data presented in the study are deposited in the (https://www.ncbi.nlm.nih.gov/geo/query/acc.cgi?acc=GSE164744) repository, accession number: GSE164744.

## Ethics Statement

The studies involving human participants were reviewed and approved by the Sixth Hospital Affiliated with Shanghai Jiao Tong University. The patients/participants provided their written informed consent to participate in this study.

## Author Contributions

ZZ and C-kC started the project, and conceived and designed the experiments. YJ, FX, and L-xS performed the experiments. LS and JG analyzed the data. C-kC took the primary responsibility for the final content. ZZ, YJ, FX, L-xS, LS, JG, and C-kC read and approved the final manuscript. All authors contributed to the article and approved the submitted version.

## Conflict of Interest

The authors declare that the research was conducted in the absence of any commercial or financial relationships that could be construed as a potential conflict of interest.

## Footnotes

1 http://www.webgestalt.org/option.php

2 http://string-db.org
