# Supplementary material for: Decitabine Induces Change of Biological Traits in Myelodysplastic Syndromes via FOXO1 Activation
Source: Front Genet. 2021 Jan 27;11:603956. doi: 10.3389/fgene.2020.603956 (PMC7873873; doi:10.3389/fgene.2020.603956)
Supplement: Supplementary file 1 [file Data_Sheet_1.docx]

**Supplementary**

**Table1. Characteristic of MDS patients**

| **No** | **Gender** | **Age** | **Blast(%)** | **karyotype** | **Diagnose** | **IPSS-R** | **status** | **2016 IWG Response Criteria** |
| --- | --- | --- | --- | --- | --- | --- | --- | --- |
| 1* | female | 63 | 12.2 | 46,XX,del(5)(q31),del(7)(q22)[6]/46,XX[10] | MDS-EB-2 | 7 | primary | PR |
| 2* | female | 67 | 14.5 | 46,XY/46,XY,dup(1)(q11q32),del(20)(q11)[15] | MDS-EB-2 | 6.5 | primary | mCR |
| 3* | male | 82 | 10.8 | 46,XY,t(3;9)(q21;p24)[20] | MDS-EB-2 | 6.5 | primary | CR |
| 4 | female | 69 | 13.5 | 46,XX[20] | MDS-EB-2 | 6.5 | primary | CR |
| 5 | male | 70 | 16 | 46,XY,+1,der(1;7)(q10;p10)[20] | MDS-EB-2 | 7 | primary |  |
| 6 | female | 63 | 13 | 46,XX[20] | MDS-EB-2 | 5.5 | primary | PR |
| 7 | male | 59 | 12.6 | 49,XX,+21[16] /46,XY[4] | MDS-EB-2 | 7 | primary | HI |
| 8 | male | 71 | 7.6 | 47,XY,+8[16]/46,XY[2] | MDS-EB-1 | 5.5 | primary | mCR |
| 9 | female | 73 | 14.2 | 47,XY,+8[15]/46,XY[5] | MDS-EB-2 | 6.5 | primary | PR |
| 10 | male | 68 | 8.5 | 46,XX[20] | MDS-EB-1 | 5.5 | primary | HI |
| 11 | male | 74 | 11 | 46,XX,+8,+9[15]/46,XY[5] | MDS-EB-2 | 6.5 | primary | SD |
| 12 | female | 75 | 16.3 | 48,XX,18,19[15]/46,XX[5] | MDS-EB-2 | 7 | primary | mCR |

**Abbreviations:** IPSS-R, Revised International Prognostic Scoring System; IWG, International Working Group; ORR, overall response rate; CR, complete response; mCR, marrow CR; PR, partial response; HI, hematologic improvement; SD, stable disease; PD, progressive disease; NR, no response. MDS-EB-2, MDS with excess blasts (MDS-EB-2).

*means for RT^2^ Profiler PCR Arrays.

**S. Table 2 Primary antibodies for WB**

| **Antibody** | **Catalogue number** | **Sources** |
| --- | --- | --- |
| FOXO1 | #2880 | Cell Signaling Technology |
| pFOXO1(Ser256) | #9461 | Cell Signaling Technology |
| Bim | #2880 | Cell Signaling Technology |
| Puma | #60434 | Cell Signaling Technology |
| FasL | #24633 | Cell Signaling Technology |
| CDKN1A | #2947 | Cell Signaling Technology |
| CDKN1B | #3686 | Cell Signaling Technology |
| CCND1 | #2922 | Cell Signaling Technology |
| CCND2 | #3741 | Cell Signaling Technology |
| PTEN | #9188 | Cell Signaling Technology |
| pPI3K(p85) | SAB4502195 | Sigma‑Aldrich, Merck KGaA |
| pAKT(Ser473) | sc293125 | Santa Cruz Biotechnology |
| TLR4 | #14358 | Cell Signaling Technology |
| pSTAT1 (phospho Y701) | Ab29045 | Abcam |
| pSTAT3(phospho Y705) | Ab76315 | Abcam |
| T-bet | ab91109 | Abcam |
| GAPDH | #8884 | Cell Signaling Technology |

**S. Table 3. List of genes involved in Innate and Adaptive Immunity**

|  | **Gene symbol** | **Name** | **Description** |
| --- | --- | --- | --- |
| **1** | *APCS* | Amyloid P component, serum | II, IR |
| **2** | *C3* | Complement component 3 | II, HI, IR |
| **3** | *CASP1* | Caspase 1, apoptosis-related cysteine peptidase (interleukin 1, beta, convertase) | II |
| **4** | *CCL2* | Chemokine (C-C motif) ligand 2 | II (Cytokine), AI (Cytokine), HI |
| **5** | *CCL5* | Chemokine (C-C motif) ligand 5 | II (Cytokine), AI (Cytokine), IR |
| **6** | *CCR4* | Chemokine (C-C motif) receptor 4 | AI (Th2 marker, Treg marker) |
| **7** | *CCR5* | Chemokine (C-C motif) receptor 5 | AI (Th1 marker) |
| **8** | *CCR6* | Chemokine (C-C motif) receptor 6 | AI (Th17 marker), HI |
| **9** | *CCR8* | Chemokine (C-C motif) receptor 8 | AI (Treg marker) |
| **10** | *CD14* | CD14 molecule | II |
| **11** | *CD4* | CD4 molecule | II, AI, DRV |
| **12** | *CD40* | CD40 molecule, TNF receptor superfamily member 5 | II, AI, DRV |
| **13** | *CD40LG* | CD40 ligand | II, AI |
| **14** | *CD80* | CD80 molecule | AI (Th1 marker, T Cell activation) |
| **15** | *CD86* | CD86 molecule | AI (Th2 marker, T Cell activation), DRV |
| **16** | *CD8A* | CD8a molecule | II, AI, DRV |
| **17** | *CRP* | C-reactive protein, pentraxin-related | II, AI, HI, IR |
| **18** | *CSF2* | Colony stimulating factor 2 (granulocyte-macrophage) | II (Cytokine), AI (Cytokine) |
| **19** | *CXCL10* | Chemokine (C-X-C motif) ligand 10 | II (Cytokine), AI (Cytokine), DRV |
| **20** | *CXCR3* | Chemokine (C-X-C motif) receptor 3 | AI (Th1 marker) |
| **21** | *DDX58* | DEAD (Asp-Glu-Ala-Asp) box polypeptide 58 | II (PRR), DRV |
| **22** | *FASLG* | Fas ligand (TNF superfamily, member 6) | AI |
| **23** | *FOXP3* | Forkhead box P3 | AI (Treg marker), IR |
| **24** | *GATA3* | GATA binding protein 3 | AI (Th2 marker) |
| **25** | *HLA-A* | Major histocompatibility complex, class I, A | II, AI, DRV |
| **26** | *HLA-E* | Major histocompatibility complex, class I, E | II |
| **27** | *ICAM1* | Intercellular adhesion molecule 1 | AI (T Cell activation) |
| **28** | *IFNA1* | Interferon, alpha 1 | II (Cytokine), AI (Cytokine) |
| **29** | *IFNAR1* | Interferon (alpha, beta and omega) receptor 1 | II, DRV |
| **30** | *IFNB1* | Interferon, beta 1, fibroblast | II (Cytokine), AI (Th2 marker), HI, DRB, DRV |
| **31** | *IFNG* | Interferon, gamma | AI (Th1 marker, T Cell activation, cytokine), HI, DRB |
| **32** | *IFNGR1* | Interferon gamma receptor 1 | AI |
| **33** | *IL10* | Interleukin 10 | AI (Th2 marker, Treg marker, cytokine) |
| **34** | *IL13* | Interleukin 13 | AI (Th2 marker, cytokine) |
| **35** | *IL17A* | Interleukin 17A | AI (Th17 marker, cytokine) |
| **36** | *IL18* | Interleukin 18 (interferon-gamma-inducing factor) | II (Cytokine), AI (Th1 marker, Th2 marker, cytokine) |
| **37** | *IL1A* | Interleukin 1, alpha | II (Cytokine), IR |
| **38** | *IL1B* | Interleukin 1, beta | II (Cytokine), AI, IR |
| **39** | *IL1R1* | Interleukin 1 receptor, type I | II, AI |
| **40** | *IL2* | Interleukin 2 | II (Cytokine), AI (Cytokine) |
| **41** | *IL23A* | Interleukin 23, alpha subunit p19 | AI (Th1 marker, T Cell activation, cytokine), DRB, DRB |
| **42** | *IL4* | Interleukin 4 | AI (Th2 marker, cytokine), IR |
| **43** | *IL5* | Interleukin 5 (colony-stimulating factor, eosinophil) | AI (Th2 marker, cytokine) |
| **44** | *IL6* | Interleukin 6 (interferon, beta 2) | AI (Th2 marker, T Cell activation, cytokine), HI, IR, DRB, DRV |
| **45** | *CXCL8* | Interleukin 8 | II (Cytokine), AI (Cytokine) |
| **46** | *IRAK1* | Interleukin-1 receptor-associated kinase 1 | II |
| **47** | *IRF3* | Interferon regulatory factor 3 | II, AI, DRV |
| **48** | *IRF7* | Interferon regulatory factor 7 | II, AI |
| **49** | *ITGAM* | Integrin, alpha M (complement component 3 receptor 3 subunit) | II, AI |
| **50** | *JAK2* | Janus kinase 2 | AI |
| **51** | *LY96* | Lymphocyte antigen 96 | II |
| **52** | *LYZ* | Lysozyme | II, DRB |
| **53** | *MAPK1* | Mitogen-activated protein kinase 1 | II |
| **54** | *MAPK8* | Mitogen-activated protein kinase 8 | II, AI |
| **55** | *MBL2* | Mannose-binding lectin (protein C) 2, soluble | II, AI, HI, IR, DRB |
| **56** | *MPO* | Myeloperoxidase | II |
| **57** | *MX1* | Myxovirus (influenza virus) resistance 1, interferon-inducible protein p78 (mouse) | II, AI |
| **58** | *MYD88* | Myeloid differentiation primary response gene (88) | II, DRB |
| **59** | *NFKB1* | Nuclear factor of kappa light polypeptide gene enhancer in B-cells 1 | II, AI |
| **60** | *NFKBIA* | Nuclear factor of kappa light polypeptide gene enhancer in B-cells inhibitor, alpha | II |
| **61** | *NLRP3* | NLR family, pyrin domain containing 3 | II (PRR), DRV |
| **62** | *NOD1* | Nucleotide-binding oligomerization domain containing 1 | II (PRR), DRV |
| **63** | *NOD2* | Nucleotide-binding oligomerization domain containing 2 | II (PRR), AI (Th2 marker), HI, DRV |
| **64** | *RAG1* | Recombination activating gene 1 | AI |
| **65** | *RORC* | RAR-related orphan receptor C | AI (Th17 marker) |
| **66** | *SLC11A1* | Solute carrier family 11 (proton-coupled divalent metal ion transporters), member 1 | AI (Th1 marker, T Cell activation), DRB |
| **67** | *STAT1* | Signal transducer and activator of transcription 1, 91kDa | II, AI |
| **68** | *STAT3* | Signal transducer and activator of transcription 3 (acute-phase response factor) | AI (Th17 marker), IR |
| **69** | *STAT4* | Signal transducer and activator of transcription 4 | AI (Th1 marker) |
| **70** | *STAT6* | Signal transducer and activator of transcription 6, interleukin-4 induced | AI (Th2 marker) |
| **71** | *TBX21* | T-box 21 | AI (Th1 marker) |
| **72** | *TICAM1* | Toll-like receptor adaptor molecule 1 | II, DRV |
| **73** | *TLR1* | Toll-like receptor 1 | II (PRR), DRB |
| **74** | *TLR2* | Toll-like receptor 2 | II (PRR) |
| **75** | *TLR3* | Toll-like receptor 3 | II (PRR), DRB, DRV |
| **76** | *TLR4* | Toll-like receptor 4 | II (PRR), AI (Th1 marker), DRB |
| **77** | *TLR5* | Toll-like receptor 5 | II (PRR) |
| **78** | *TLR6* | Toll-like receptor 6 | II (PRR), AI (Th1 marker), DRB |
| **79** | *TLR7* | Toll-like receptor 7 | II (PRR), DRV |
| **80** | *TLR8* | Toll-like receptor 8 | II (PRR), DRV |
| **81** | *TLR9* | Toll-like receptor 9 | II (PRR), DRB |
| **82** | *TNF* | Tumor necrosis factor | II (Cytokine), AI (Cytokine), HI, IR |
| **83** | *TRAF6* | TNF receptor-associated factor 6 | II |
| **84** | *TYK2* | Tyrosine kinase 2 | DRV |

II (Innate Immunity); AI (Adaptive Immunity); HI (Humoral Immunity); IR (Inflammatory Response); DRB (Defense Response to Bacteria); DRV (Defense Response to Viruses); PRR (Pattern Recognition Receptor).

**S. Table 4. Primers sequences used for Quantitative real-time PCR**

| Gene | Primer sequence |
| --- | --- |
| GAPHD | forward:5’-AACTTTGGCATTGTGGAAGG-3’ |
|  | reverse:5’-ACACATTGGGGGTAGGAACA-3’ |
| T-bet | forward: 5′- GGTTGCGGAGACATGCTGA-3′ |
|  | reverse: 5′- GTAGGCGTAGGCTCCAAGG-3′ |
| STAT1 | forward: 5’-GTGGAAAGACAGCCCTGCAT-3’ |
|  | reverse:5’-ACTGGACCCCTGTCTTCAAGAC-3’ |
| PD-1 | forward: 5’-ACCTGGGTGTTGGGAGGGCA-3’ |
|  | reverse: 5’- GGAGTGGATAGGCCACGGCG-3’ |
| PDL-1 | forward: 5’-TGGCATTTGCTGAACGCATTT-3’ |
|  | reverse:5’-TGCAGCCAGGTCTAATTGTTTT-3’ |
